# Striking increase in incidence of prostate cancer in men aged < 60 years without improvement in prognosis

**DOI:** 10.1038/sj.bjc.6690004

**Published:** 1999-09-24

**Authors:** P N Post, D Stockton, T W Davies, J-W W Coebergh

**Affiliations:** Comprehensive Cancer Centre South, PO Box 231, Eindhoven 5600 AE, The Netherlands; Department of Epidemiology & Biostatistics, Erasmus University Rotterdam, PO Box 1738, Rotterdam 3000 DR, The Netherlands; East Anglian Cancer Intelligence Unit, Department of Community Medicine, Institute of Public Health, University of Cambridge, Robinson Way, Cambridge CB2 2SR, UK

**Keywords:** prostatic neoplasm, incidence, prognosis, mortality, middle age

## Abstract

Increased awareness and improved diagnostic techniques have led to earlier diagnosis of prostate cancer and increased detection of subclinical cases, resulting in improved prognosis. We postulated that the considerable increase in incidence under age 60 is not attributable only to increased detection. To test this hypothesis, we studied incidence, mortality and relative survival among middle-aged patients diagnosed in south-east Netherlands and East Anglia (UK) between 1971 and 1994. Prostate-specific antigen (PSA) testing did not occur before 1990. Between 1971 and 1989, the age-standardized incidence at ages40–59 increased from 8.8 to 12.5 per 10^5^ in The Netherlands and from 7.0 to 11.6 per 10^5^ in East Anglia.Five-year relative survival did not improve in East Anglia and even declined in south-east Netherlands from 65% [95% confidence interval (CI) 47–83) in 1975–79 to 48% (CI 34–62) in 1985–89. Mortality due to prostate cancer among men aged 45–64 years increased by 50% in south-east Netherlands and by 61% in East Anglia between 1971 and 1989, but decreased slightly in the 1990s. Because other factors adversely influencing the prognosis are unlikely, our results indicate an increase in the incidence of fatal prostate cancer among younger men in the era preceding PSA testing. © 1999 Cancer Research Campaign


*British Journal of Cancer* (1999) **79**(1), 13–17
© 1999 Cancer Research Campaign

### Striking increase in incidence of prostate cancer in men aged < 60 years without improvement in prognosis

**PN Post[1,2], D Stockton[3], TW Davies[3] and J-WW Coebergh[1,2]**

[1]Comprehensive Cancer Centre South, PO Box 231, 5600 AE Eindhoven, The Netherlands; [2]Department of Epidemiology & Biostatistics, Erasmus University Rotterdam, PO Box 1738, 3000 DR Rotterdam, The Netherlands; [3]East Anglian Cancer Intelligence Unit, Department of Community Medicine, Institute of Public Health, University of Cambridge, University Forvie Site, Robinson Way, Cambridge CB2 2SR, UK

**Summary** Increased awareness and improved diagnostic techniques have led to earlier diagnosis of prostate cancer and increased detection of subclinical cases, resulting in improved prognosis. We postulated that the considerable increase in incidence under age 60 is not attributable only to increased detection. To test this hypothesis, we studied incidence, mortality and relative survival among middle-aged patients diagnosed in south-east Netherlands and East Anglia (UK) between 1971 and 1994. Prostate-specific antigen (PSA) testing did not occur before 1990. Between 1971 and 1989, the age-standardized incidence at ages 40–59 increased from 8.8 to 12.5 per $10^5$ in The Netherlands and from 7.0 to 11.6 per $10^5$ in East Anglia. Five-year relative survival did not improve in East Anglia and even declined in south-east Netherlands from 65% [95% confidence interval (CI) 47–83) in 1975–79 to 48% (CI 34–62) in 1985–89. Mortality due to prostate cancer among men aged 45–64 years increased by 50% in south-east Netherlands and by 61% in East Anglia between 1971 and 1989, but decreased slightly in the 1990s. Because other factors adversely influencing the prognosis are unlikely, our results indicate an increase in the incidence of fatal prostate cancer among younger men in the era preceding PSA testing.

**Keywords**: prostatic neoplasm; incidence; prognosis; mortality; middle age

Worldwide, prostate cancer has been diagnosed with increasing frequency over recent decades (Coleman et al, 1993). This increase is partly attributable to increased application of transurethral resection of the prostate (TURP) (Potosky et al, 1990) for treating benign prostatic hyperplasia (BPH), which results in the incidental detection of subclinical prostate cancer in approximately 10% of cases (Rohr, 1987). More recently, case finding by prostate-specific antigen (PSA) testing has resulted in a further increase in incidence (Potosky et al, 1995). As a consequence, the prognosis for prostate cancer patients has improved in many countries (Levi et al, 1992; Black et al, 1993; Kosary et al, 1995; Helgesen et al, 1996), reflecting the increasing proportion detected at a preclinical stage. In south-east Netherlands, overall 5-year relative survival improved modestly from 57% in 1970–1979 to 61% in 1987–1992 (Coebergh et al, 1995); however, the improvement occurred largely in elderly patients. The highest increase in incidence was observed in the 1980s in the youngest age groups in south-east Netherlands; in men under age 65 (Post et al, 1998). As TURP is about seven times less frequently used for men under age 60 than in men over age 75 (SIG, 1997) because of the lower prevalence of BPH (Chute et al, 1993) and the much lower incidence of cancer, it seemed that the increase in incidence at younger ages might not be caused by a higher detection rate. We studied trends in the incidence and survival of patients with prostate cancer aged 40–59 years in south-east Netherlands and, for comparison, in East Anglia, UK, which has a similar system of data collection and a more or less comparable system of health care provision. We also studied trends in mortality due to prostate cancer and analysed changes in the distribution of grade, stage and initial treatment. While the main study period (1971–89) was before the introduction of PSA testing, data for 1990–1994 are also included to provide some insight into more recent trends.

*Received 9 January 1998*
*Revised 26 June 1998*
*Accepted 30 June 1998*

*Correspondence to:* PN Post

### MATERIALS AND METHODS

### Study population

We used data from two cancer registries, the Eindhoven Cancer Registry in south-east Netherlands and the East Anglian Cancer Registry in the UK. In both registries, most cases were identified from pathology reports, which are routinely sent to the registries; the remainder were reported by medical record departments in the regional hospitals and the regional radiotherapy institute (Eindhoven) or the district general hospitals (East Anglia). South-east Netherlands has a population of almost 1 million inhabitants and is characterized by good access to specialized medical care provided in eight large community hospitals. National data show that TURP was increasingly used between 1970 and 1990 and was the main treatment modality for both cancer of the prostate and BPH in the 1970s. Radiotherapy was applied increasingly after 1980, but radical prostatectomy was rare. PSA assessment was not introduced until 1990. In the Eindhoven Registry, the vital status of all cases was determined through municipal civil registries until 1 April 1994. Five patients (2.8%) were lost to follow-up before this date (mostly due to repeatedly moving home), and so were censored in the analysis. East Anglia has a population of around 2.2 million inhabitants and has three specialist hospitals with oncology centres and a further six district general hospitals. The majority of the population lives in and around the three major cities of Cambridge, Ipswich and Norwich, where they have good access to specialized medical care. PSA assays were introduced in 1991. The East Anglian Registry receives notification of the deaths of all individuals flagged as having cancer or where cancer is mentioned on the death certificate, from the Office of National Statistics. In addition, it actively follows up its patients 3 years after diagnosis and then every 5 years until death, ensuring almost complete follow-up. Mortality data were obtained from Statistics Netherlands and the British Office of National Statistics. The mid-year population estimates were used for each individual year included in the study.

### Analysis

The incidence rates per 100 000 person–years for the age band 40–59 were standardized to the European Standard Population using 5-year age-specific rates for 5-year calendar periods. As the median survival time is approximately 5 years, we calculated the age-standardized mortality rates for the age band 45–64 years. Poisson regression analysis was applied to model incidence and mortality (Clayton and Hills, 1993), using the GENMOD procedure of the statistical package SAS. Significance of terms in the models was tested with the likelihood-ratio test (Clayton and Hills, 1993). Crude and relative survival rates were calculated using the actuarial (life table) method. Relative survival is the ratio of the crude to the expected survival (Ederer et al, 1961). The expected survival was calculated from life tables derived from regional mortality statistics; data were compiled into 5-year age groups and calendar year. A software package from the Finnish Cancer Registry (Hakulinen and Abeywickrama, 1985) was used to calculate the survival rates. The rates were adjusted for the changing age distribution of the patient groups during the course of the follow-up (Ederer II option). Cases identified at death were excluded from the analyses. We used grade information as it was registered, that is scored according to the classification of malignant tumours (UICC, 1992). Information about stage was available only in the Eindhoven Cancer Registry and only from 1980. Based on clinical TNM assessment (UICC, 1992), we classified stages in three categories: small tumours confined to the prostate (T1–T2) without evidence of metastases were classified as localized; tumours that invaded surrounding structures (T3–T4) but without evidence of metastases were classified as locally advanced; patients with distant or lymph node metastases were classified as metastasized. Grade and stage, both available from 1980, are presented both with and without inclusion of the unknown cases. Differences in proportions were tested with the chi-square test (excluding unknown cases).

We classified initial treatment as TURP (including patients detected incidentally because of TURP), hormonal treatment, hormonal treatment after TURP and radiotherapy. Patients receiving radiotherapy after TURP or radiotherapy and hormonal therapy were included in the radiotherapy group. Treatment information was available for the period 1971–89 in the Eindhoven Registry and from 1980 to 1989 in East Anglia.

### RESULTS

The number of patients aged 40–59 diagnosed with prostate cancer between 1971 and 1989 was 181 in south-east Netherlands and 384 in East Anglia, being 7% and 4% respectively of patients at all ages with prostate cancer diagnosed between 1971 and 1989. The proportion of patients with a histologically confirmed diagnosis at ages 40–59 was more than 95% during the whole study period in both populations.

Between 1971 and 1989, the age-adjusted incidence rate for men aged 40–59 increased from 8.8 per $10^5$ to 12.5 per $10^5$ in south-east Netherlands and from 7.0 per $10^5$ to 11.6 per $10^5$ in East Anglia (Figure 1). The mean age at diagnosis of the patients in this age group barely changed over the study period, being 55.4 years in The Netherlands and 55.6 in East Anglia. A multivariate model for the incidence up to 1994 was fitted that included age group, registry and calendar period [deviance 35.0, 34 degrees of freedom (d.f.)]. The risk ratio of the incidence increased for each subsequent period up to 1990–94 (Table 1). The test for trend was significant ($P$ = 0.0001) and the trend was similar in both registries. A similar result was obtained when the period 1990–94 was excluded. The age-standardized mortality rate for prostate cancer among men aged 45–64 years increased between 1971 and 1989 from 7.4 to 11.1 per $10^5$ in south-east Netherlands and from 7.5 to 12.1 per $10^5$ in East Anglia. A model was built including age group, calendar period and registry (deviance 47.1, 34 d.f.). The risk ratio increased with each subsequent period up to 1985–89, followed by a slight decline in 1990–94 (Table 1). Nevertheless,

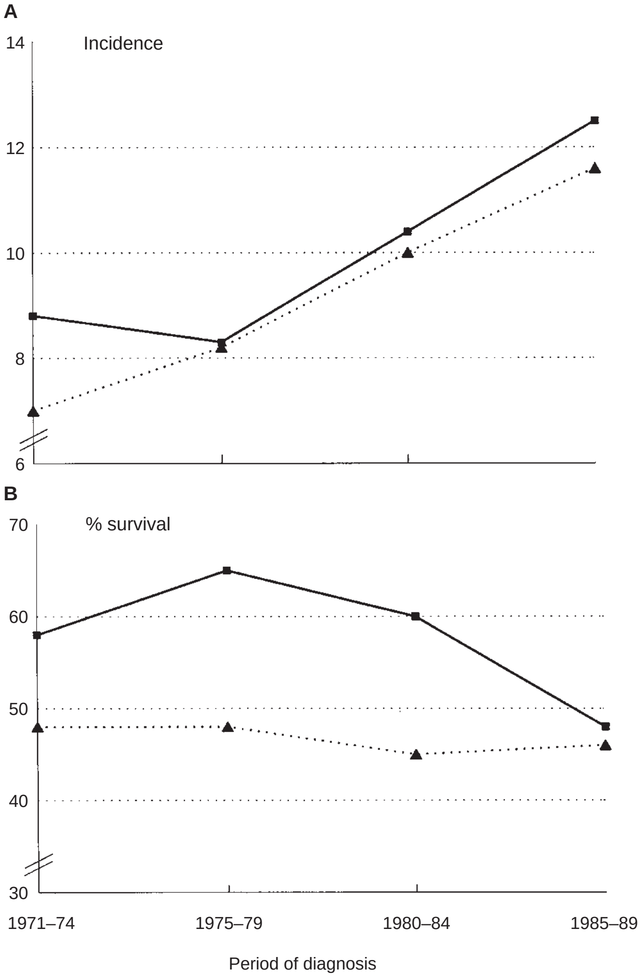

**Figure 1** Incidence rates per 100 000 person–years standardized to the European standard population (**A**) and 5-year relative survival rates (**B**) for prostate cancer patients aged 40–59 in south-east Netherlands (■) and East Anglia (▲)

 *© Cancer Research Campaign 1999*

**Table 1** Risk ratios and 95% confidence intervals for incidence (40–59 years) of and mortality (45–64 years) due to prostate cancer in south-east Netherlands and East Anglia

| | Incidence (40–59 years) | | Mortality (45–64 years) | |
|---|---|---|---|---|
| | **Risk ratio (95% CI)** | **No. of cases** | **Risk ratio (95% CI)** | **No. of cases** |
| 1971–74 (reference) | 1 | 82 | 1 | 82 |
| 1975–79 | 1.11 (0.84–1.47) | 127 | 1.02 (0.77–1.36) | 108 |
| 1980–84 | 1.33 (1.02–1.73) | 162 | 1.24 (0.95–1.63) | 140 |
| 1985–89 | 1.53 (1.19–1.99) | 194 | 1.63 (1.26–2.12) | 195 |
| 1990–94 | 1.91 (1.49–2.46) | 255 | 1.53 (1.18–1.98) | 191 |
| *P*-value trend | 0.0001 | | 0.0001 | |

**Table 2** Trend in stage distribution (%) (with and without unknown cases) of prostate cancer patients aged 40–59 in south-east Netherlands, 1980–94

| | **1980–84** | **1985–89** | **1990–94** |
|---|---|---|---|
| *n* | 51 | 65 | 108 |
| | % within period | % | % |
| Stage | | | |
| Localized | 40 | 53 | 57 |
| Locally advanced | 8 | 12 | 12 |
| Metastasized | 39 | 29 | 18 |
| Unknown | 13 | 6 | 13 |
| Stage | | | |
| Localized | 47 | 56 | 66 |
| Locally advanced | 9 | 13 | 13 |
| Metastasized | 44 | 31 | 21 |
| *P*-value $\chi^2$ | 0.07 | | |

the test for trend was significant ($P$ = 0.0001) and the trend was similar in both registries.

In south-east Netherlands, 5-year relative survival improved slightly in the early 1970s, but declined from 65% (95% CI 47–83) in 1975–79 to 48% (CI 34–62) in 1985–89 (Figure 1). In East Anglia, 5-year relative survival was initially considerably lower, being 48% (CI 34–62) in 1971–74 and decreasing slightly to 46% (CI 36–56) in 1985–89. The crude survival followed a similar trend.

In spite of an increase in the estimated proportion of patients with localized cancer from 47% in 1980–84 to 56% in 1985–89 in south-east Netherlands (Table 2), the estimated proportion of patients with poorly differentiated tumours increased from 15% to 25% (Table 3). The proportion of patients aged 40–59 years receiving radiotherapy increased from 21% in 1975–79 to 55% in 1985–89 in south-east Netherlands and radiotherapy has also been the main treatment modality in East Anglia between 1980 and 1989 (Table 4). The remainder of patients received endocrine therapy or TURP. Radical prostatectomy was only rarely applied before 1990 in both populations.

### DISCUSSION

We report a similar rise in the incidence of prostate cancer among men aged 40–59 years in south-east Netherlands and East Anglia and no improvement in prognosis in the period preceding the introduction of PSA testing in 1990. Improved diagnosis does not seem to be an important factor in the rise in incidence because it would have resulted in the inclusion of more non-aggressive cases and, thus, in improved survival. Moreover, in spite of a more favourable stage distribution, we did not observe an increase in well-differentiated tumours in this period.

Our findings are conditional on the accuracy of the two cancer registries. Comparisons with mortality data and analysis of referral patterns indicate that both registries can be considered virtually complete for prostate cancer as of 1971 and both comply with the standards of the International Agency for Research on Cancer (Parkin et al, 1992). Few patients were lost to follow-up, so that selective loss is not likely to be an issue. The study populations were relatively small, especially in south-east Netherlands, but our

**Table 3** Trend in grade distribution (%) (with and without unknown cases) of prostate cancer patients aged 40–59 in south-east Netherlands and East Anglia, 1980–94

| | South-east Netherlands | | | East Anglia | | |
|---|---|---|---|---|---|---|
| | **1980–84** | **1985–89** | **1990–94** | **1980–84** | **1985–89** | **1990–94** |
| *n* | 51 | 65 | 108 | 111 | 129 | 147 |
| | % within period | % | % | % | % | % |
| Differentiation | | | | | | |
| Well | 36 | 34 | 41 | 24 | 22 | 25 |
| Moderately | 31 | 37 | 31 | 18 | 24 | 29 |
| Poorly | 12 | 23 | 22 | 18 | 21 | 22 |
| Unknown | 20 | 6 | 6 | 40 | 33 | 24 |
| Differentiation | | | | | | |
| Well | 46 | 36 | 44 | 40 | 33 | 33 |
| Moderately | 39 | 39 | 33 | 30 | 36 | 38 |
| Poorly | 15 | 25 | 23 | 30 | 31 | 29 |
| *P*-value $\chi^2$ | | 0.6 | | | $P > 0.1$ | |

© Cancer Research Campaign 1999 

**Table 4** Trend in initial treatment (%) of prostate cancer patients aged 40–59 in south-east Netherlands and East Anglia

| | 1971–74 | 1975–79 | 1980–84 | 1985–89 |
|---|---|---|---|---|
| *Eindhoven* | | | | |
| Radiotherapy | 0 | 21 | 38 | 55 |
| Endocrine therapy | 32 | 24 | 4 | 22 |
| Endocrine + TURP | 36 | 7 | 13 | 9 |
| TURP | 28 | 48 | 40 | 14 |
| None | 4 | | 5 | |
| *East Anglia* | | | | |
| Radiotherapy | | | 42 | 46 |
| Endocrine therapy | | | 12 | 6 |
| Endocrine + TURP | | | 15 | 15 |
| TURP | | | 26 | 26 |
| None/Unknown | | | 5 | 7 |

findings are not compatible with a significant improvement in survival. Moreover, registry-based studies in other countries have provided similar results. In Sweden, 5-year relative survival for prostate cancer patients aged 45–54 years improved from 42% in the early 1960s to 62% in the late 1970s but declined to 50% in the early 1980s (Helgesen et al, 1996). In Scotland, it declined from 47% in 1978–82 to 32% in 1983–87 (Black et al, 1993). In Switzerland (Vaud), the relative survival for patients aged under 60 only improved slightly from 39% in 1974–78 to 41% in 1979–83 (Levi et al, 1992), which was similar to the situation for Finnish patients (Dickman et al, 1998). We do not know why the prognosis barely changed in East Anglia but deteriorated in south-east Netherlands. A lower level of access to specialized care may have played a role in the initially lower survival in East Anglia, because the Eurocare study showed that similar differences in survival existed between south-east Netherlands and Great Britain for patients with lung, breast or colorectal cancer, which may be related to differences in stage at diagnosis (Berrino et al, 1995). Increasing awareness of prostate cancer and early diagnosis was probably not apparent before 1980 in East Anglia.

Our hypothesis, that a genuine increase in incidence has occurred, is also supported by the increase in mortality attributable to prostate cancer under 65 years, which was similar in both populations, although it was followed by a small decline in 1990–94. An analysis of national mortality data from 1950–89 showed an increase in mortality due to prostate cancer in consecutive birth cohorts of men born around 1925 in The Netherlands (Van der Gulden et al, 1994). In Norway, the increase in both incidence and mortality due to prostate cancer between 1957 and 1991 was highest in men under 60 years (Harvei et al, 1996). In the USA, mortality due to prostate cancer has started to decline since 1991–95, in particular for men under age 75 (Hoeksema and Law, 1996), whereas it had increased slightly in those under age 65 in the years beforehand (Kosary et al, 1995). The decline, however, may be related to the widespread introduction of early detection and intervention techniques. Changes in cause-specific mortality such as that observed for cancer of the prostate in Eindhoven and East Anglia should be interpreted with caution. All-cause mortality has also been declining in both populations for the age group studied. In particular, mortality attributable to cardiovascular causes declined in The Netherlands from 499 per $10^5$ in 1970 to 301 per $10^5$ in 1990 for men aged 45–64 years (Central Bureau of Statistics, 1992). As a result of the decrease in concurrent causes of death, the probability that prostate cancer was recorded as the cause of death may have increased. However, this explanation would be more plausible for mortality in older age groups.

We suggest, therefore, that an increased risk of prostate cancer in those under age 60 has almost certainly occurred over the period 1971 to 1989. As far as we know, only one aetiological study has focused on the under 60 age group, reporting a relative risk (RR) of 1.9 for cigarette smoking, a RR of 1.4 for vasectomy and a RR of 2.3 for early age at first sexual intercourse (Honda et al, 1988). Recently, Rodriguez et al (1997) reported a significant association of current smoking with fatal prostate cancer (RR 1.34), which was highest among men below 60 years (RR 1.83), but there was no association with the number of cigarettes smoked or with the duration of smoking at baseline for the cohort in 1982. Nor was there any increased risk for former smokers (Rodriguez et al, 1997). This, as well as results from other large studies, suggests that smoking may adversely influence survival in prostate cancer patients. Increased occurrence of a factor associated with a worse survival could be an alternative explanation for our findings. However, smoking is not a likely candidate, because the proportion of male smokers decreased markedly from 95% in 1960 to 40% in 1981 in The Netherlands (Janssen-Heijnen et al, 1995) and also in England (Coleman et al, 1993).

Unfavourable changes in the health care system do not seem to play a role, because the proportion of cases detected at an earlier stage increased over the study period, at least in south-east Netherlands. Furthermore, radiotherapy was applied increasingly during the study period. Although a beneficial effect of radiotherapy on survival has not been proven definitively (Lu-Yao and Yao, 1997), it seems unlikely that radiotherapy has been detrimental for prostate cancer patients. We, therefore, assume that increased incidence of fatal prostate cancer, of which the cause still needs to be unravelled, should explain our findings.

Although the incidence continued to increase, mortality due to prostate cancer decreased slightly in the 1990s. This could mean that the postulated genuine increase in incidence has come to a halt in the 1990s. Continuing studies of incidence and survival may provide more insight into the nature of the most recent increase in incidence.

From the current study, we conclude that increased detection of prostate cancer by TURP cannot explain the considerable increase in incidence between 1971 and 1989 in the age group below 60 years.

© Cancer Research Campaign 1999

### ACKNOWLEDGEMENTS

This study received financial support from the Comprehensive Cancer Centre South (IKZ) and the Netherlands Institute for Health Sciences (NIHES). We thank the registry personnel for registration of the data.

### REFERENCES

Berrino F, Sant M, Verdecchia A, Capocaccia R, Hakulinen T and Esteve J (1995) *Survival of Cancer Patients in Europe. The Eurocare Study*. Publication no 132. IARC: Lyon

Black RJ, Sharp L and Kendrick SW (1993) *Trends in Cancer Survival in Scotland 1968–1990*, pp. 46–47. National Health Service Scotland: Edinburgh

Central Bureau of Statistics (1992) *Mortality by Some Main Causes of Death*, 1970–1990. SDU: The Hague

Chute CG, Panser LA, Girman CJ, Oesterling JE, Guess HA, Jacobsen SJ and Lieber MM (1993) The prevalence of prostatism: a population-based survey of urinary symptoms. *J Urol* **150**: 85–89

Clayton D and Hills M (1993) *Statistical Models in Epidemiology*. Oxford University Press: New York

Coebergh JWW, van der Heijden LH and Janssen-Heijnen MLG (eds) (1995) *Cancer Incidence and Survival in the Southeast of the Netherlands, 1955–1994: a Report from the Eindhoven Cancer Registry*, p 101. Comprehensive Cancer Centre South: Eindhoven

Coleman MP, Esteve J, Damiecki P, Arslan A and Renard H (1993) *Trends in Cancer Incidence and Mortality*, Scientific publication no. 121, pp. 499–520. IARC: Lyon

Dickman PW, Hakulinen T, Luostarinen T, Pukkala E, Sankila R, Söderman B and Tippo L (1998) Survival of cancer patients in Finland 1955–1994. *Acta Oncol* (in press)

Ederer F, Axtell LM and Cutler SJ (1961) The relative survival rate: a statistical methodology. *Natl Cancer Inst Monogr* **6**: 101–121

Hakulinen T and Abeywickrama KH (1985) A computer package for relative survival analysis. *Comp Progr Biomed* **19**: 197–207

Harvei S, Tretli S and Langmark F (1996) Cancer of the prostate in Norway 1957–1991 – a descriptive study. *Eur J Cancer* **32A**: 111–117

Helgesen F, Holmberg L, Johansson JE, Bergström R and Adami HO (1996) Trend in prostate cancer survival in Sweden, 1960 through 1988: evidence of increasing diagnosis of nonlethal tumours. *J Natl Cancer Inst* **88**: 1216–1221

Hoeksema MJ and Law C (1996) Cancer mortality rates fall: a turning point for the nation (news). *J Natl Cancer Inst* **88**: 1706–1707

Honda GD, Bernstein L, Ross RK, Greenland S, Gerkins V and Henderson BE (1988) Vasectomy, cigarette smoking, and age at first sexual intercourse as risk factor for prostate cancer in middle-aged men. *Br J Cancer* **57**: 326–331

Janssen-Heijnen MLG, Nab HW, van Reek J, van der Heijden LH, Schipper R and Coebergh JWW (1995) Striking changes in smoking behaviour and lung cancer incidence by histological type in Southeast Netherlands, 1960–1991. *Eur J Cancer* **31A**: 949–952

Kosary CL, Ries LAG, Miller BA, Hankey BF and Edwards BK (eds) (1995) *SEER Cancer Statistics Review, 1973–1992: Tables and Graphs*, p. 395. NIH publication no. 96-2789. National Cancer Institute: Bethesda

Levi F, Randimbison L, Van-Cong T, Franceschi S and La Vecchia C (1992) Trends in cancer survival in Vaud, Switzerland. *Eur J Cancer* **28A**: 1490–1495

Lu-Yao GL and Yao S-L (1997) Population-based study of long-term survival in patients with clinically localised prostate cancer. *Lancet* **349**: 906–910

Parkin DM, Muir CS, Whelan SL, Gao YT, Ferlay J and Powell J (1992) *Cancer Incidence in Five Continents*, Vol. VI. IARC scientific publication no. 120. IARC: Lyon

Post PN, Kil PJM, Crommelin MA, Schapers RFM and Coebergh JWW (1998) Trends in incidence and mortality rates for prostate cancer before and after prostate-specific antigen introduction: a registry-based study in Southeastern Netherlands, 1971–1995. *Eur J Cancer* **34**: 705–709

Potosky AL, Kessler L, Gridley G, Brown CC and Horm JW (1990) Rise in prostatic cancer incidence associated with increased use of transurethral resection. *J Natl Cancer Inst* **82**: 1624–1628

Potosky AL, Miller BA, Albertsen PC and Kramer BS (1995) The role of increasing detection in the rising incidence of prostate cancer. *JAMA* **273**: 548–552

Rodriguez C, Tatham LM, Thun M, Calle EE and Health CW (1997) Smoking and fatal prostate cancer in a large cohort of adult men. *Am J Epidemiol* **145**: 466–475

Rohr LR (1987) Incidental adenocarcinoma in transurethral resections of the prostate. *Am J Surg Pathol* **11**: 53–58

SIG Health Care Information (1997) *Trends in Urology 1986–1995*. SIG Health Care Information:Utrecht

Van der Gulden JWJ, Kiemeney LALM, Verbeek ALM and Straatman H (1994) Mortality trend from prostate cancer in the Netherlands (1950–1989). *Prostate* **24**: 33–38

UICC (1992) *TNM Atlas Illustrated Guide to the TNM/pTNM Classification of Malignant Tumours*, 4th edn, 2nd revision, pp. 141–144. Springer: Berlin

*© Cancer Research Campaign 1999*
